# Bioaccessible arsenic in soil of thermal areas of Viterbo, Central Italy: implications for human health risk

**DOI:** 10.1007/s10653-021-00914-1

**Published:** 2021-04-21

**Authors:** V. Rimondi, P. Costagliola, P. Lattanzi, T. Catelani, S. Fornasaro, D. Medas, G. Morelli, M. Paolieri

**Affiliations:** 1 Dipartimento di Scienze della Terra, Università di Firenze, Via G. La Pira 4, 50121 Florence, Italy; 2 CNR- IGG, Via G. La Pira 4, 50121 Florence, Italy; 3 Piattaforma di Microscopia, Università di Milano-Bicocca, Piazza della Scienza 2, 20126 Milan, Italy; 4 Dipartimento di Scienze Chimiche e Geologiche, Università di Cagliari, Cittadella Universitaria di Monserrato - Blocco A, S.S. 554 bivio per Sestu, 09042 Monserrato, CA Italy

**Keywords:** Arsenic, Bioaccessibility, Calcite, Geogenic, Soil ingestions, Thermal springs

## Abstract

Thermal waters near the city of Viterbo (Central Italy) are known to show high As contents (up to 600 µg/l). Travertine is precipitated by these waters, forming extended plateau. In this study, we determine the As content, speciation and bioaccessibility in soil and travertine samples collected near a recreational area highly frequented by local inhabitants and tourists to investigate the risk of As exposure through accidental ingestion of soil particles. (Pseudo)total contents in the studied soils range from 17 to 528 mg/kg, being higher in soil developed on a travertine substrate (197 ± 127 mg/kg) than on volcanic rocks (37 ± 13 mg/kg). In travertines, most As is bound to the carbonatic fraction, whereas in soil the semimetal is mostly associated with the oxide and residual fractions. Accordingly, bioaccessibility (defined here by the simplified bioaccessibility extraction test, SBET; Oomen et al., 2002.) is maximum (up to 139 mg/kg) for soil developed on a travertine substrate, indicating a control of calcite dissolution on As bioaccessibility. On the other hand, risk analysis suggests a moderate carcinogenic risk associated with accidental soil ingestion, while dermal contact is negligible. By contrast, ingestion of thermal water implies a higher carcinogenic and systemic health risk.

**Supplementary Information:**

The online version contains supplementary material available at 10.1007/s10653-021-00914-1.

## Introduction

Arsenic (As) contamination of drinking water, air, food and beverages is a major global health issue, affecting more than 300 million people worldwide (Quansah et al., 2015). The consequences of exposure to As for human health are severe, ranging from dermatologic manifestations to carcinogenic and systemic non-carcinogenic effects.

Geothermal As is common in active and former continental-volcanic settings such as in New Zealand, the Andes, Southern Italy, and to a lesser extent in oceanic-volcanic terrains (Ravenscroft et al., 2009). Besides, As spreads in the environment as a consequence of changing redox conditions, which trigger As mobilization from adsorbing mineral phases. This is especially testified in floodplains and/or rice paddy fields, which commonly undergo flooding/non-flooding conditions during their agricultural seasons (e.g., Kim et al., 2021). Arsenic contents of uncontaminated soils worldwide range from 1 to 100 mg/kg, but in general As levels are mostly below 10 mg/kg, and often below 5 mg/kg (Ravenscroft et al., 2009). As indicated by the EuroGeoSurveys database (Salminen et al., 2005), As is unevenly distributed among topsoils of European countries, displaying significant enrichment in southern Europe (Italy, France and Spain) with respect to Scandinavia (10.5–2.3 mg/kg, respectively), reflecting the soil finer nature and the long weathering history (Reimann et al., 2009). The As enrichment in soils is indeed considered one of the causes of the higher incidence of dementia in some European countries, among others Italy, France and Spain (Dani, 2010).

Ingestion of contaminated drinking water and food is the primary route to As exposure for humans (Polya & Lawson, 2016). However, incidental ingestion of As-contaminated soil is a significant exposure pathway through hand-to-mouth transfer during outdoor activities. This is especially true for children (2–6 years old; Calabrese et al., 1989; Kwon et al., 2004), who ingest soil both deliberately and involuntarily by putting dirty hands and objects in their mouths, and are hence exposed to the risk associated with this behavior (Ljung et al., 2007). Once ingested, the risk for human health is associated with As bioavailability, defined as the fraction of an ingested dose that crosses the gastrointestinal epithelium and becomes available for absorption by internal tissues (US EPA, 2007a). Bioavailability can be determined by in vivo studies (Bradham et al., 2018). In the last decades, As bioaccessibility, i.e., the As fraction soluble in the gastrointestinal tract and available for absorption (Rodriguez et al., 1999; Ruby et al., 1999; US EPA, 2007a), is often employed as a surrogate of bioavailability. To estimate bioaccessibility, in vitro simulations of gastrointestinal fluids are carried on. For this purpose, different extractant methods have been established (Bradham et al., 2018 and reference therein). Among them, the simplified bioaccessibility extraction test (SBET) (Oomen et al., 2002; US EPA, 2007b), which simulates the action of gastric juices in a single step, has been extensively used by many researchers (Bagherifam et al., 2014; Mingot et al., 2011; Smith et al., 2008), and validated with in vivo tests (Juhasz et al., 2014).

Linear regression analysis and hierarchical modeling employing soil physicochemical properties, such as the soil elemental composition, pH, mineralogy, particle size, soil aging, have been frequently employed to predict As bioaccessibility (Appleton et al., 2012; Cave et al., 2003; Juhasz et al., 2007; Karna et al., 2017; Martínez-Sánchez et al., 2013; Nelson et al., 2018). On the other hand, selective/sequential chemical extractions (SEC) and synchrotron radiation investigations (Kim et al., 2014; Mikutta et al., 2014) can be used to unravel As solid phase speciation, identifying the mineral pools which contribute at most of the bioaccessible As from the in vitro tests. Moreover, the knowledge of the solid phases hosting bioaccessible As helps predicting the environmental processes which enhance As bioaccessibility through destabilization of the mineral carrier phases. In soils, Fe oxy(hydr)oxides have the prominent role in controlling As bioaccessibility (Hiller et al., 2018). Specifically, amorphous and poorly crystalline Fe oxy(hydr)oxides increase As bioaccessibility, whereas crystalline phases reduce it (Girouard & Zagury, 2009; Kim et al., 2014; Mikutta et al., 2014; Palumbo-Roe et al., 2015; Smith et al., 2008; Whitacre et al., 2013).

To date, most studies were conducted on soils with low carbonate contents. Carbonates, and especially calcite, may, however, trap significant amounts of As in natural environments (e.g., Costagliola et al., 2013), possibly controlling As bioaccessibility.

In Italy, hotspots of As occur in Central Italy, especially between Tuscany (Benvenuti et al., 2009; Costagliola et al., 2010; Morelli et al., 2017) and Latium, particularly in the province of Viterbo. Here groundwaters from the volcanic aquifer exceed the thresholds established by the European Drinking Water Directive 98/83/EC. Public awareness of the problem grew during the second half of 2010 and led to a widespread use of bottled water for drinking purposes. There is, however, poor awareness of the risk deriving from exposure to As associated with the thermal springs of Viterbo (Central Italy), which are well known to discharge waters rich in As (Cinti et al., 2019, and references therein). These waters also deposit travertine (Pentecost, 1995), which has formed an extended plateau. Little information is, however, available on As distribution in the carbonate rock itself and in the surrounding soils. In this paper, As distribution is investigated in travertine deposits and soils surrounding these thermal springs; the main goals are: i) to define As distribution in soils in relation to the different geological substrata; ii) to quantify As bioaccessibility and how it is correlated with As fractionation, determined by SBET and sequential extraction procedures, respectively; iii) to estimate the risk associated with the accidental soil ingestion among adults and children in the area.

## Study area

**Fig. 1:**
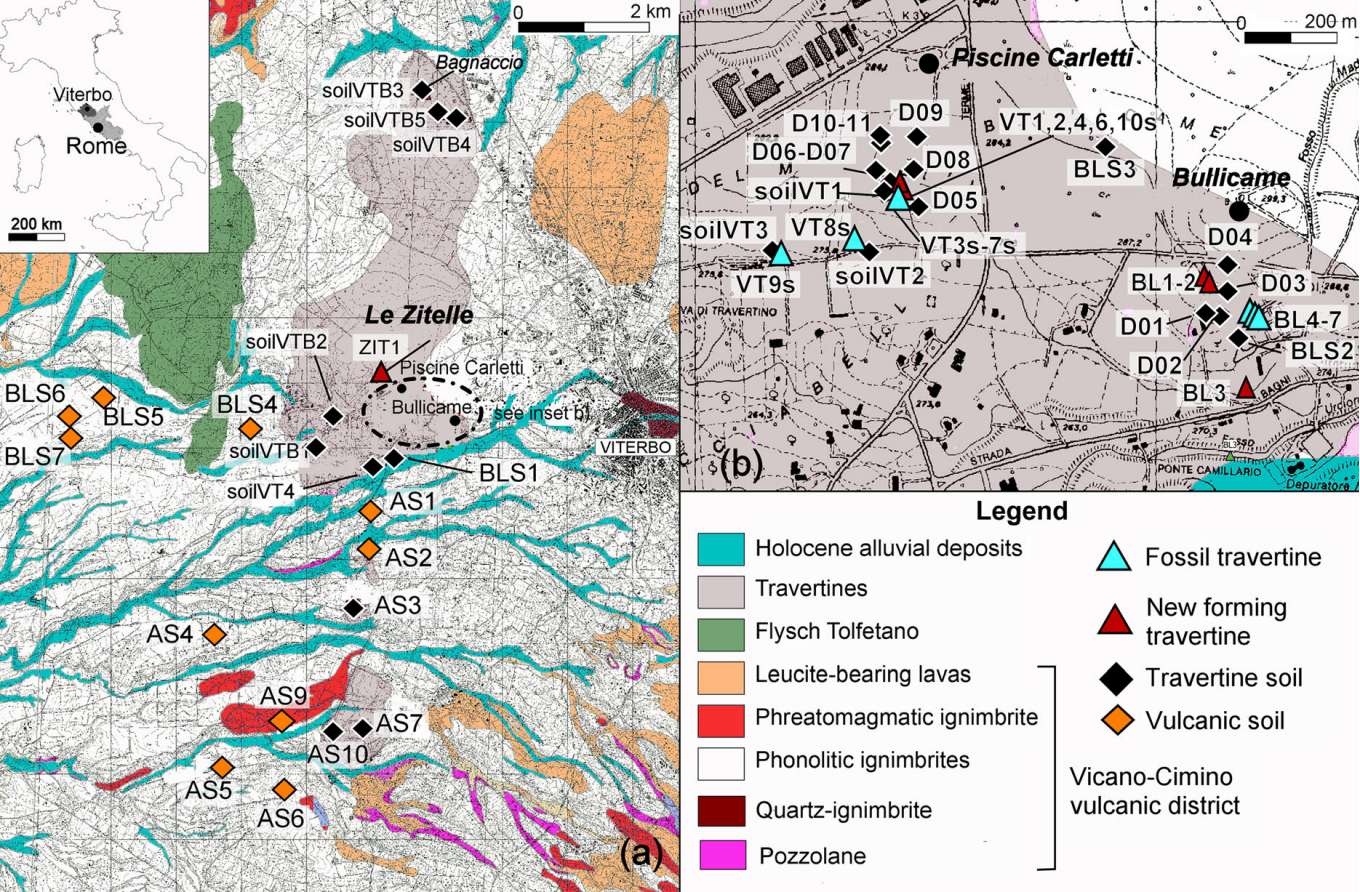
**a** Geological map of the Viterbo area and location of samples; the dash-point circled area is enlarged in **b**; **b** detailed map of thermal hot-spring sites of Piscine Carletti and Bullicame

The study area is located few km west of the city of Viterbo (northern Latium), in the surroundings of the city thermal area (Fig. 1). Geologically, it belongs to the Vicano-Cimino volcanic complex extending between the Tyrrhenian coast and the Apennines chain. Plio-Quaternary extensional tectonics related to the post-collisional phases of Apennines orogeny were responsible for crustal thinning (< 25 km; Scrocca et al., 2003), heat flow anomalies (100–200 mW/m^2^; Della Vedova et al., 2001), and the development of subduction-related magmatism (Peccerillo, 2017, and references therein). Specifically, the Vico complex (0.4–0.1 Ma), consisting of volcanic rocks belonging to the Roman Magmatic Province, diffusely crops out in the study area. It consists of silica-oversaturated to silica-undersaturated potassic and ultrapotassic magmas, which partially superimpose on the Mt. Cimini rocks (~ 1.3–0.9 Ma) belonging to the Tuscan Magmatic Province. The pre-volcanic substratum is composed by Neogene marine and continental deposits, which filled NW–SE-oriented grabens (Barberi et al., 1994), Upper Cretaceous-Oligocene Flysch (Ligurian Units), Triassic-Paleogene carbonate rocks and evaporites (Tuscan-Marche Units), and Cenozoic turbidite deposits (Flysch units). The volcanic rocks are covered by Pleistocene-Holocene continental deposits and Holocene travertines, which extend horizontally and sub-horizontally forming vast plateau (Manfra et al., 1976). According to Minissale et al. (2002), the formation of travertines is associated with decarbonation processes affecting Mesozoic units at depth.

In the proximity of the study area, two aquifers were recognized: i) a shallow circulation within the volcanic rocks and ii) a deep circulation confined in the Triassic-Paleogene carbonate rocks, which hosts a thermal reservoir, characterized by high As contents (176–371 µg/l; Angelone et al., 2009). These two aquifers are separated by low-permeability horizons (Flysch Units and/or Pliocene–Pleistocene sedimentary rocks), but interact both vertically and laterally (Baiocchi et al., 2012). West of Viterbo, the reduced thickness of the Flysch units and faulting allows the uprising of thermal waters (Angelone et al., 2009). Hot (54–60 °C) H_2_S-rich spring waters emerge along a N-S fault at Bagnaccio, Piscine Carletti, Bullicame and Le Zitelle, where travertine is being deposited in stream beds.

The hot springs close to the city of Viterbo are well known since at least III century B.C. by the Etruscans, when thermal waters were already being used for therapeutic properties. Nowadays, natural springs are employed in the therapies of a variety of medical complaints. The recreational area of Viterbo is visited by thousands of people during the whole year, including children (Strangio and Teodori, 2015). In particular, the Bullicame (also referred as Bullicame west; Pentecost, 1995) and Piscine Carletti (also referred as Bullicame 3; Duchi et al., 1985) sites rise up in a large green area freely open to the public. Here, the waters were artificially channeled to provide bathing water for visitors and local inhabitants. It is a common practice for bathers to spread the white carbonate mud on skin lesions, inhale H_2_S, or drink the mineral waters, which are believed to have healthy effects.

## Materials and methods

### Sampling

Samples were collected inside and around the hot springs of Bullicame and Piscine Carletti (Fig. 1a, b) from soils formed on top of the main geological formations cropping out in the area, as verified in the field. In total, 10 soils developed on volcanic rocks and 26 on travertine were sampled (see Table S1 for GPS coordinates). Some of them were collected from agricultural fields, mainly cultivated for wheat. After the removal of vegetation, wherever present, about 1 kg of topsoil (0–10 cm) was collected with a Teflon scoop. Soils are classified as Luvic Endoleptic Phaeozems according to the cartography of Latium Region (Napoli et al., 2019), where additional details on soil main characteristics may be found.

Additionally, travertine mud (i.e., currently precipitating from waters, “new forming” thereafter) and rocky (“fossil”) travertine were sampled (*n* = 17) in three locations (Le Zitelle, Bullicame and Piscine Carletti).

### Chemical and mineralogical analysis

Travertine and soil samples were dried at room temperature for one month and then disaggregated with a ceramic mortar. Soils were sieved in two granulometric fractions: (1) the < 2 mm fraction was used for (pseudo) total metal characterization, according to the Italian law requirement (D.L. 152/2006) and for SEC, and (2) the < 250 µm fraction was primarily used for the SBET procedure and for (pseudo) total metal concentrations in samples analyzed for SBET. Representative samples (fossil travertine (*n* = 4), new-forming travertine (*n* = 6), travertine soils (*n* = 7), and volcanic soils (*n* = 5)) were analyzed for the whole chemistry by wavelength-dispersive X-ray fluorescence (WD XRF) with a PHILIPS PW 1480.

Loss on ignition (LOI) was measured by gravimetric methods; 0.50 g of oven-dried (at 105 °C overnight) samples were heated to 950 °C in quartz-fiber crucibles for 2 h. The LOI values were employed for major elements calculations (Franzini et al., 1975). The analytical quality was controlled by using international standards, and the relative differences between the results and certified values are less than 5%.

X-ray diffraction (XRD) patterns were collected on the < 250 µm fraction of selected soil samples and travertines with a Philips PW 1050/37 instrument, operating with a Cu anode and a graphite monochromator, driven by a PANalytical X’Pert PRO data acquisition system.

All analyses were performed at the Dipartimento di Scienze della Terra, Università di Firenze (Italy).

#### Aqua regia digestion

Samples dissolution (on the < 2 mm fraction) was performed by digestion with aqua regia (HCl/HNO_3_ 3:1) to quantify the pseudo-total As (As_T-2000_) concentrations in soils and travertines (*n* = 53). As stated before, the < 250 µm soil fraction analyzed for SBET was also digested by aqua regia to determine pseudo-total As (As_T-250_). Digestions were accomplished both by the US EPA 3051 method by microwave digestion (Milestone CEM MARS 6) in pre-cleaned Teflon vessels at 175 °C for 20 min and in a sand bath at 50 °C for 3 h. The last procedure was employed to avoid contamination of Teflon bombs for samples with high As content. The obtained solutions were then filtered to 0.45 µm after adequate cooling, diluted to 100 ml volume with Milli-Q water, stored in polyethylene bottles, and later analyzed by inductively coupled plasma optical emission spectroscopy coupled with hydride generation (HG-ICP-OES; PerkinElmer Optima 8000) within 1 week from digestion. Accuracy was evaluated with international standards (Montana Soil, 2711, RTS4, 2710) and internal laboratory standards (among them, a travertine rock). Recovery was between 90 and 100%. Samples were processed together with blanks prepared with the same acid mixture to evaluate potential contamination from the reagents and sample containers. Instrumental detection limits for ICP-OES were < 0.3 µg/l.

Reproducibility was checked by duplicate analyses of six random selected samples, and differences were less than 15%.

#### Simplified bioaccessibility extraction test: SBET

Soils (*n* = 14, underlined in Table 1) with different As contents (high, low and medium values of the dataset) developed on travertine substratum were selected for the in vitro SBET procedure to quantify the bioaccessible As fraction (As_SBET_) (Oomen et al., 2002; Ruby et al., 1999; US EPA, 2012). Around 1 ± 0.05 g of < 250 μm soil particle size fraction was mixed with a solution of 50 mL of glycine (0.4 M) at pH = 1.50 ± 0.05 and adjusted with HCl (37% v/v). This mixture was rotated end-over-end at 30 rpm for 1 h at 37 °C. pH was controlled every 5 min (pH acceptable variation should be ± 0.5) and adjusted whenever necessary. The mixture was centrifuged, the supernatant was separated with nitrate cellulose filters (0.45 µm), and it was preserved at 4 °C until analysis. The concentration of As was determined by atomic absorption spectroscopy (AAS) coupled with hydride generation (PerkinElmer Analyst 100). Quality controls involved preparation and analysis of three sample triplicates on six samples (D01, D03, D06, AS10, SOILVTB4 and SOILVT2), and on blank solutions. The relative standard deviation of the replicate analyses was < 4%. Arsenic contents in blank solutions were below the detection limit (10 µg/L).

**Table 1:** Arsenic contents (mg/kg) of the investigated soils and travertines

Sample ID	Sample type	Location	As_T-2000_	As_T-250_
soilVT1	Trav soil	Piscine Carletti	323	nd
soilVT2	Trav soil	Agricultural area	495	569
soilVT3	Trav soil	Agricultural area	321	nd
soilVT4	Trav soil	Agricultural area	81	nd
BLS 1	Trav soil	Agricultural area	47	nd
BLS 2	Trav soil	Agricultural area	90	nd
BLS 3	Trav soil	Agricultural area	42	nd
soilVTB1	Trav soil	Agricultural area	195	nd
soilVTB2	Trav soil	Agricultural area	58	nd
soilVTB3	Trav soil	Agricultural area	132	nd
soilVTB4	Trav soil	Agricultural area	130	104
soilVTB5	Trav soil	Agricultural area	119	nd
AS3	Trav soil	Agricultural area	255^a^	nd
AS7	Trav soil	Agricultural area	528	512
AS10	Trav soil	Agricultural area	420	518
D01	Trav soil	Bullicame	150^a^	135
D02	Trav soil	Bullicame	152	158
D03	Trav soil	Bullicame	166^a^	151
D04	Trav soil	Bullicame	141	124
D05	Trav soil	Piscine Carletti	164	169
D06	Trav soil	Piscine Carletti	246	252
D07	Trav soil	Piscine Carletti	211	224
D08	Trav soil	Piscine Carletti	186	182
D09	Trav soil	Piscine Carletti	173	166
D10	Trav soil	Piscine Carletti	155	147
D11	Trav soil	Piscine Carletti	152	nd
BLS4	Vulc soil	Agricultural area	56^a^	nd
BLS5	Vulc soil	Agricultural area	30	nd
BLS6	Vulc soil	Agricultural area	32	nd
BLS7	Vulc soil	Agricultural area	17^a^	nd
AS1	Vulc soil	Agricultural area	26	nd
AS2	Vulc soil	Agricultural area	36	nd
AS4	Vulc soil	Agricultural area	24	nd
AS5	Vulc soil	Agricultural area	53	nd
AS6	Vulc soil	Agricultural area	48	nd
AS9	Vulc soil	Agricultural area	43	nd
ZIT1	New-form trav	Le Zitelle	73	nd
BL1	New-form trav	Bullicame	154	nd
BL2	New-form trav	Bullicame	206	nd
BL3	New-form trav	Bullicame	201	nd
BL4	Fossil trav	Bullicame	160	nd
BL5	Fossil trav	Bullicame	186^a^	nd
BL7	Fossil trav	Bullicame	123	nd
VT1s	New-form trav	Piscine Carletti	130	nd
VT2s	New-form trav	Piscine Carletti	80	nd
VT3s	Fossil trav	Piscine Carletti	195	nd
VT4s	New-form trav	Piscine Carletti	164	nd
VT5s	New-form trav	Piscine Carletti	148	nd
VT6s	New-form trav	Piscine Carletti	168	nd
VT7s	Fossil trav	Piscine Carletti	50	nd
VT8s	Fossil trav	Piscine Carletti	39	nd
VT9s	Fossil trav	Piscine Carletti	276	nd
VT10s	New-form trav	Piscine Carletti	125	nd

^a^Mean of triplicate analysis; reproducibility < 4 mg/kg As

Underlined samples are those investigated for SBET

nd stands for not determined

Relative bioaccessibility—RBA (%)—was calculated as (Hu et al., 2011; Juhasz et al., 2007):

1 $${\text{RBA}} \left( \% \right) = \frac{{{\text{As}}_{\text{SBET}} }}{{{\text{As}}_{\text{T - 250}} }} \times 100,$$

where As_SBET_ and As_T-250_ refer to As extracted from SBET and aqua regia digestions (250 µm), respectively.

#### Sequential extraction scheme: SEC

The As content of specific geochemical fractions of travertine soils can be extracted selectively by using appropriate reagents, and it was here quantified through two SEC procedures. Details of the reagents employed, the solid-to-liquid ratios and extractions times for each As fraction (As non-specifically bound—A_SO4_; As specifically bound—A_PO4_; As associated with carbonates—A_CARB_; As associated with amorphous Fe oxides—A_OX_; As in the residual fraction—A_AR_) are reported in Table 2. The first scheme (*A*), made of six extraction steps (*A*1–*A*6) and modified after Wenzel et al. (2001), was preliminarily applied to four travertines and four soils to screen the main As-hosting phase. Following the preliminary results, to selectively quantify the amount of As associated with carbonates with respect to other mineral phases as a whole, a simplified four-step scheme A* (Costagliola et al., 2013) was applied to other six soil samples. Here, the aqua regia digestion (*A*_AR_*) was hence directly applied after the step devoted to carbonates (*A*_CARB_*). Therefore, the sum of As extracted from *A*_OX_ to *A*_AR_ ($\sum {\text{As}} _{{A_{\text{OX}} - A_{\text{AR}} }} )$ of scheme A should be comparable to the As extracted from As_AR_* of scheme A*. For the sake of simplicity, in the following we will report $\left( {\sum {\text{As}} _{{A_{\text{OX}} - A_{\text{AR}} }} } \right)$ as *A*_AR_^SUM^, so that it can be directly compared with *A*_AR_*.

**Table 2:** Summary of the sequential chemical extraction procedures (A and A*) employed in this study

Fractions		Extractant	Extractions conditions	SSR**	Behavioral classes
Scheme A	Scheme A*				
A_SO4_	A_SO4_*	0.05 mol/L (NH_4_)_2_SO_4_	4 h shaking, 25 °C	1:25	Non-specifically sorbed
A_PO4_	A_PO4_*	0.05 mol/L (NH_4_)_2_PO_4_	16 h shaking, 25 °C	1:25	Specifically sorbed
A_CARB_	A_CARB_*	40 ml of 1 mol/l sodium acetate/acetic acid buffer; pH 5	12 h shaking, 25 °C	1:25	As bound to carbonates
A_OX_	nd	0.2 mol/L NH4-oxalate buffer; pH 3.25	4 h shaking in the dark, 25 °C	1:25	As bound to amorphous Fe oxides
A_OX+C_	nd	0.2 mol/L NH4-oxalate buffer + 0.1 M ascorbic acid; pH 3.25	30 min in a sand basin at 96 °C	1:25	As bound to crystalline Fe oxides
A_AR_	A_AR_*	aqua regia; HCl/HNO_3_ 3:1	3 h in sand bath, 50 °C for scheme A; microwave assisted for scheme A*	1:20	Residual

**Solid-to-liquid ratio

The sum of As extracted from A_OX_ to A_AR_ ($\sum {\text{As}} _{{A_{\text{OX}} - A_{\text{AR}} }}$) of the scheme A is comparable to A_AR_* of the scheme A* (nd stands for not determined)

Extractions were conducted by weighing 1 ± 0.05 g of sample in 50 ml vials and sequentially adding 20–25 ml of the respective extraction solution (Table 2). After each step, the suspension was centrifuged at 10,000 g for 15 min, and the supernatant was separated. Only for step A_CARB_* the residue was filtered through 0.45-µm paper filter (Whatman 42), and the filter microwave digested. The concentration of As was determined by AAS coupled with hydride generation (PerkinElmer Analyst 100).

The analytical quality of the sequential extraction was controlled calculating the As recovery (R %) as:

$${\text{As }}\,{\text{recovery }}\left( \% \right) = \sum {{\text{As}}_{\text{SEC}} /{\text{As}}_{\text{T}} \times \, 100}$$

where ΣAs_SEC_ is the sum of As extracted from each single extraction step and As_T_ is the As extracted from the bulk sample with aqua regia. Recovery was between 84 and 122%. Reproducibility was evaluated by analyzing three samples (D06, D07 and soilVT2) in duplicate. Differences are < 15% for all extraction steps.

#### Exposure and human health risk assessment

Humans exposure to As in the thermal areas of Viterbo may occur via two principal contact routes with contaminated soils and waters, which are (1) oral ingestion and (2) dermal absorption. Based on the data of the present study, we specifically evaluated the human health risk, following the procedure by USEPA (1989) and transposed by the Italian legislation (D.L. 152/2006 and D.L. 4/2008), associated with accidental ingestion of contaminated soils by residents visiting the thermal pools. Moreover, we evaluated the health risk associated with: (1) the oral exposure derived from the voluntary ingestion of thermal waters for depurative purpose; (2) the dermal exposure associated with water (during bathing activities) and soils (including voluntary applications on skin of thermal muds). The cumulative effect of the multiple exposure pathways was hence calculated (see later). Children (age 1–6) and adults’ exposure scenarios were considered.

Average daily dose (ADD) (or lifetime average daily dose, LADD), expressed in mg/kg day of As from ingestion of soil (ADD_s,ing_) or dermal contact (ADD_s,derm_) was calculated with the following formula (US EPA, 1989, 1992, 2004):

2 $${\text{ADD}}_{\text{s,ing}} = \frac{{C_{\text{s}} \times {\text{IR}}_{s} \times {\text{EF}} \times {\text{ED}} \times {\text{CF}}_{s} }}{{{\text{BW}} \times {\text{AT}}_{\text{NC}} }}$$

3 $${\text{ADD}}_{\text{s,derm}} = \frac{{C_{\text{s}} \times {\text{SA}} \times {\text{AF}} \times {\text{ABS}} \times {\text{EF}} \times {\text{ED }} \times {\text{EV}} \times {\text{CF}}_{s} }}{{{\text{BW}} \times {\text{AT}}_{\text{NC}} }}$$

where *C*_s_ is the As content in soils in the < 250 µm fraction (As_T-250_) at each sampling site (at those sites we also have the available glycine extractable (i.e., SBET) contents (mg/kg)); IR_s_ the soil ingestion rate (mg/day); EF the exposure frequency (days/year); ED the exposure duration (years); EV the event/day; CF_s_ the unit conversion factor (10^−6^ kg/mg); BW the body weight (kg), AT_NC_ the averaging time for exposure (ED*365 days for non-carcinogenic substances), SA the skin surface area available for contact (cm^2^/event); AF the soil to skin adherence factor (mg/cm^2^); ABS the absorption factor for the skin (unitless); BW the body weight (kg). For carcinogenic chemicals, the LADD was standardly calculated by substituting AT_C_ for AT_NC_, where the averaging time corresponds to lifetime (i.e., AT = 70*365) (US EPA, 1989, 2004).

Similarly, we calculated ADD (or LADD) for water ingestion (ADD_w,ing_) or dermal contact (ADD_w,derm_):

4 $${\text{ADD}}_{\text{w, ing }} = \frac{{C_{\text{w}} \times {\text{IR}}_{\text{w }} \times {\text{EF}} \times {\text{ED}}}}{{{\text{BW}} \times {\text{AT}}_{\text{NC}} }}$$

5 $$ADD_{w, derm } = \frac{{C_{w } \times SA \times PC \times ET \times EF \times ED \times CF_{w} }}{{BW \times AT_{NC} }},$$

where *C*_w_ is chemical concentration in water (mg/l) from the Bullicame and Piscine Carletti thermal pools (Cinti et al., 2019), IR_w_ is the volume (l) of drunk thermal water during or soon after the visit to the pools, PC is the chemical-specific dermal permeability constant (cm/hr); ET is the exposure time (hours/day); CF_w_ is the volumetric conversion factor for water (1 l/1000 cm^3^), and the other variables as described above for Eqs. (2) and (3). Exposure frequency (EF = 100) was determined specifically for this study, assuming that local people visit the thermal recreational areas during the weekend (i.e., twice a week). Similarly, we assumed that the consumption of thermal waters for depurative purposes (i.e., IR_w_) does not exceed a water bottle (1 l) or a quarter of water bottle (0.25 l) in 2 days for adults and children, respectively. These estimates are obviously tentative: to our knowledge, there is no systematic survey of the actual habits of site visitors. The other specific parameters for ADD or LADD calculations are available in Table 3. For adults, ADD or LADD was age-mediated (ADD_adj_) considering a child (6 years) plus adults (24 years) exposure to As, corresponding to a total ED of 30 years.

**Table 3:** Summary of the parameters employed for the ADD and LADD (mg/kg day) calculation for soil ingestion, soil dermal, water ingestion and water dermal exposure scenarios

Parameters	Symbol	Units	Values		Source
*Soil ingestion exposure*			Children	Adults	
Element concentration in water	C_S_	mg/kg	site-specific		This study
Ingestion rate of soil (US EPA)*	IR_S_	mg/day	40	10	US EPA (2017)
Exposure frequency	EF	days/year	100		This study
Exposure duration	ED	years	6	30	US EPA (2004)
Body weight	BW	kg	15	70	US EPA (2004)
Averaging time (non-cancerogenic)	AT_NC_	days	2190	10,950	US EPA (1989)
Averaging time (carcinogenic)	AT_C_	days	25,550		US EPA (1989)
Conversion factor	CF	kg/mg	1.00E-06		US EPA (1989)

Parameters	Symbol	Units	Values		Source
*Soil dermal exposure*			Children	Adults	
Chemical concentration in soil	C_S_	mg/kg	site-specific		This study
Skin surface area**	SA	cm^2^	5700	2800	US EPA (2004)
Soil to skin adherence factor	AF	mg/cm^2^	0.2	0.07	US EPA (2004)
Absorption factor for the skin	ABS	unitless	0.03		US EPA (2004)
Exposure frequency	EF	day/year	100		This study
Exposure duration	ED	years	6	30	US EPA (2004)
Body weight	BW	kg	15	70	US EPA (1989)
Averaging time (non-cancerogenic)	AT_NC_	days	2190	10,950	US EPA (1989)
Averaging time (carcinogenic)	AT_C_	days	25,550		US EPA (1989)
Conversion factor	CF	kg/mg	1.00E−03		US EPA (1989)

Parameters	Symbol	Units	Values		Source
*Water ingestion exposure*			Children	Adults	
Element concentration in water	C_W_	mg/l	0.35		Cinti et al. (2019)
Ingestion rate of water	IR_W_	l/day	0.12	0.5	This study
Exposure frequency	EF	day/year	100		This study
Exposure duration	ED	year	6	30	US EPA (2004)
Body weight	BW	year	15	70	US EPA (2004)
Averaging time (non-cancerogenic)	AT_NC_	days	2190	10,950	US EPA (1989)
Averaging time (carcinogenic)	AT_C_	days	25,550		US EPA (1989)

Parameters	Symbol	Units	Values		Source
*Water dermal exposure*			Children	Adults	
Element concentration in water	C_W_	mg/l	0.35		Cinti et al. (2019)
Skin surface area	SA	cm^2^	6600	18,000	US EPA (2004)
Chemical-specific dermal permeability constant	PC	cm/h	1.00E−03		GSI (2011)
Exposure time***	ET	hours/day	2.6		US EPA (1989)
Exposure duration****	ED	years	6	30	US EPA (2004)
Exposure frequency	EF	day/year	100		This study
Body weight	BW	kg	15	70	US EPA (2004)
Averaging time (non-cancerogenic)	AT_NC_	days	2190	10,950	US EPA (1989)
Averaging time (carcinogenic)	AT_C_	days	25,550		US EPA (1989)
Volumetric conversion factor for water	CF_W_	l/cm^3^	1.00E−03		US EPA (1989)

*Includes soil and outdoor settled dust

**Considered total body

***For swimming

****Reasonable maximum exposure during a swimming scenario

After exposure assessment, risk characterization was delineated by integrating data about toxicity (dose/response). Arsenic is considered both a threshold and a non-threshold contaminant. For threshold contaminant, a toxic effect is expected when a certain exposure concentration is surpassed (reference dose), while for non-threshold contaminants toxic effects are shown at any level of exposure. The hazard quotient (HQ), i.e., the potential for non-carcinogenic toxicity to occur, and the cancer risk (CR), i.e., the incremental probability of developing a cancer during a lifetime, refer to the threshold and non-threshold behavior, respectively. Skin cancer risk (CR_skin_) was separately calculated for dermal and ingestion scenarios and for water and soil matrix. HQ and CR_skin_ were calculated as follows (US EPA, 2007a):

$${\text{HQ}} = \frac{{{\text{ADD}} \times {\text{RBA}}}}{\text{RfD}}$$

$$CR_{skin} = LADD \times RBA \times CSF$$

where RfD is the oral reference dose of As for assessing non-cancer health effects (0.0003 mg/kg/day; US EPA, 1991) and CFS is the As cancer slope factor (1.5 mg/kg/day for oral exposure; US EPA, 2010), while RBA is the relative bioaccessibility. For soils ingestion, RBA was specifically determined in this study at each studied point analyzed by SBET (Eq. 1; §3.2.2) and not averaged (Izquierdo et al., 2015). For water ingestion, inorganic As is almost completely (~ 95%) adsorbed in the gastrointestinal tract (US EPA, 2004); consequently, a value of 100% of the ingested dose was set up in this study. For dermal contact, a dermal absorption rate of 1% was employed as suggested by Zuzolo et al. (2020). It has to be highlighted that US EPA did not define specific RfD and CSF for dermal exposure (US EPA, 2004); therefore, oral RfD and CFS are instead here employed. A systemic health risk is not expected when HQ < 1, while if HQ > 1 there is a chance that non-carcinogenic effects may occur, with a probability which tends to increase as the value of HI increases (US EPA, 2001). For carcinogenic risk, acceptable or tolerable risk for US EPA regulatory purposes is in the range of 1 × 10^−6^ and 1 × 10^−4^ (i.e., 1 case of cancer in 1,000,000 exposed people to 1 case of cancer in 10,000 exposed people). The risk level of 1 × 10^−6^ has been considered as the point of excess cancer risk, indicating 1 per 1,000,000 chance of getting cancer by single or multiple exposure routes. The safe point for carcinogenic risks must be lower than this level. Risks surpassing 1 × 10^−4^ are unacceptable and need some sort of intervention and remediation.

Cumulative hazard quotient (HQ_TOT_) and cancer risk (CR_TOT_) were finally calculated assuming addition of adverse health effects for multiple exposure routes, i.e., ${\text{HQ}}_{\text{TOT}} = \mathop \sum \limits_{n = 1}^{i} {\text{HQ}}_{i }$ and ${\text{CR}}_{\text{skin, TOT}} = \mathop \sum \limits_{n = 1}^{i} {\text{CR}}_{i }$.

## Results

### Major chemistry and pseudo-total As

Travertines are mainly composed of CaO (mean + standard deviation; $\bar{x}$_CaO_ = 54.06 ± 1.76 wt%), with minor silica (SiO_2_ ≤ 6.22 wt%) and Fe contents (Fe_2_O_3_ ≤ 1.06 wt%) (Table S2). Al, Na, Mg, K, P, Ti, and Mn oxides rarely exceed 1 weight % (Table S1). Fossil deposits show in general lower concentrations of CaO ($\bar{x}$_CaO_ = 52.57 ± 1.92%) and higher contents of Fe_2_O_3_ ($\bar{x}$_Fe2O3_ = 0.43 ± 0.43 wt%), MnO ($\bar{x}$_MnO_ = 0.12 ± 0.13 wt%) and SiO_2_ ($\bar{x}$_SiO2_ = 2.89 ± 2.41 wt%) with respect to the new-forming ones ($\bar{x}$_CaO_ = 55.04 ± 0.66 wt%; $\bar{x}$_Fe2O3_ = 0.06 ± 0.04 wt%; $\bar{x}$_MnO_ = 0.02 ± 0.01 wt%; $\bar{x}$_SiO2_ = 0.78 ± 0.41 wt%).

Aging of travertine is hence indicated by crystallization of accessory minerals, such as Fe(Mn)-oxydroxides, commonly associated with travertine deposits (e.g., Le Guern et al., 2003) and increased SiO_2_ content probably due to silicification of the deposits. Soils developed on both geological substrata (travertine and volcanic rocks) cover a wide range of SiO_2_ (31.75–55.02 wt%), and Al_2_O_3_ (10.22–21.38 wt%) contents (Table S2). Soils formed on travertine are characterized by higher contents of CaO ($\bar{x}$_CaO_ = 11.12 ± 9.74 wt%) compared to volcanic soils ($\bar{x}$_CaO_ = 4.47 ± 0.96 wt%; $\bar{x}$_LOI_ = 7.54 ± 0.96 wt%). On the contrary, volcanic soils show higher concentrations of Fe_2_O_3_ ($\bar{x}$_Fe2O3_ = 9.93 ± 1.13 wt%), Na_2_O ($\bar{x}$_Na2O_ = 1.00 ± 0.17 wt%) and K_2_O ($\bar{x}$_K2O_ = 5.80 ± 1.04 wt%) with respect to the carbonatic ones ($\bar{x}$_Fe2O3_ = 7.84 ± 1.46 wt%; $\bar{x}$_Na2O_ = 0.45 ± 0.21 wt%; $\bar{x}$_K2O_ = 2.97 ± 0.46 wt%). Chemical characteristics of volcanic soils are consistent with the geochemistry of volcanic rocks belonging to the K-alkaline cycle of the Roman Magmatic Province (Conticelli et al., 2002). CaO (wt%) concentrations reflect the variable content of calcite, which is the only carbonate mineral in soils (XRD data, not shown). Semi-quantitative estimates by XRD indicate that Bullicame soils (sample D03) are composed almost exclusively of calcite (> 90 wt%) with minor quartz and k-feldspar; however, calcite is minor at Piscine Carletti (sample D06) and in the agricultural areas, where silicate minerals (quartz, plagioclase, k-feldspar, and clay minerals) dominate. Here, minor Fe oxides (goethite) were also detected.

Arsenic in soils (As_T-2000_, referred simply as total As thereafter) ranges between 17 and 528 mg/kg (Table 1). Travertine and volcanic soils display mean total As of 197 ± 127 mg/kg and 37 ± 13 mg/kg, respectively. Irrespective of the substratum, both soil types show higher As contents compared to As upper crustal range (2–5.7 mg/kg; Wedepohl, 1995; Hu & Gao, 2008) and As in other geothermal manifestations in volcanic settings (Kusatsu, Japan: 15–170 mg/kg, Kikawada et al., 2008; Bagno Vignoni, Mt. Amiata: 4.2–344 mg/kg Chiarantini et al., 2016). Similarly, travertine rocks display hundreds of mg/kg of As (up to 276 mg/kg As; Table 1). Fossil travertines exhibit the greatest variability in As concentrations, ranging from 30 to 276 mg/kg. In the sieved fraction < 250 µm, As_T-250_ is comparable to As_T-2000_ (differences ranging between 3 and 23%).

### SEC and SBET

Results of As extraction by SEC are shown in Table 4. Specific and non-specific exchangers (steps A_SO4_ and A_PO4_) extract moderate amounts of As (As ≤ 27 mg/kg) from new-forming travertine and fossil travertine deposits. Major differences between groups are observed in the other extraction steps. In the new-forming travertine, the highest amounts of extracted As (128–162 mg/kg As) are observed in the carbonatic step (A_CARB_), while only 2–3 mg/kg As are associated with residual phases (A_OX-AR_^SUM^; Table 4).

**Table 4:** Arsenic contents (mg/kg) extracted from SEC steps on soils and travertines

Sample type	Sample ID	A_SO4-_ (A_SO4_*)	A_PO4_ (A_PO4_*)	A_CARB_ (A_CARB*_)	A_OX_	A_OX+C_	A_AR_ (A_AR_*)	A_OX-AR_^SUM^	ΣAs_SEC_
		mg/kg							
New-forming travertine	VT1s	15	13	128	2	<DL	<DL	2	158
	VT6s	4	17	162	1	1	1	3	186
Fossil travertine	VT7s	2	8	35	10	1	<DL	11	56
	VT9s	8	27	101	111	54	4	169	305
Travertine soil	D03	2	10	141	nd	nd	8	nd	161
	D06	3	14	18	nd	nd	218	nd	252
	D07	3	18	15	nd	nd	166	nd	202
	soilVT1	2	46	15	138	133	81	352	415
	soilVT2	2	45	11	nd	nd	442	nd	499
	soilVTB4	2	25	10	nd	nd	82	nd	119
	AS7	2	46	12	nd	nd	483	nd	543
	AS10	2	34	14	nd	nd	301	nd	351
	soilVT3	2	38	5	120	176	16	312	357
	soilVT4	2	7	19	31	20	75	126	154

nd stands for not determined

On the contrary, in fossil travertine, represented by sample VT9s, steps *A*_OX_, *A*_ox+C_ and *A*_AR_ extract 169 mg/kg (A_OX-AR_^SUM^ = 169 mg/kg), with 66% of As extracted in the step dedicated to amorphous Fe oxides (*A*_ox_).

Sample VT7s shows an intermediate behavior between fossil and new-forming travertines, with the highest As concentration extracted from A_CARB_ (35 mg/kg), but displaying higher As concentrations (up to 10 mg/kg) from steps *A*_OX_ to *A*_AR_ compared to new-forming travertines.

In soils, As amounts recovered from the A_SO4_ in all samples are < 5 mg/kg, while As ranges in the A_PO4_ fraction are of 10–46 mg/kg. In all samples but D03 (Bullicame), As extracted from the carbonatic step is comprised in a narrow range of 10–20 mg/kg, whereas the highest recovery is for the residual step (82–483 mg/kg) (Table 4). Notably, sample D03 is characterized by up to 141 mg/kg of As in the A_CARB_ fraction, and low recovery in A_AR_ (8 mg/kg), comparable to travertine rocks.

**Fig. 2:**
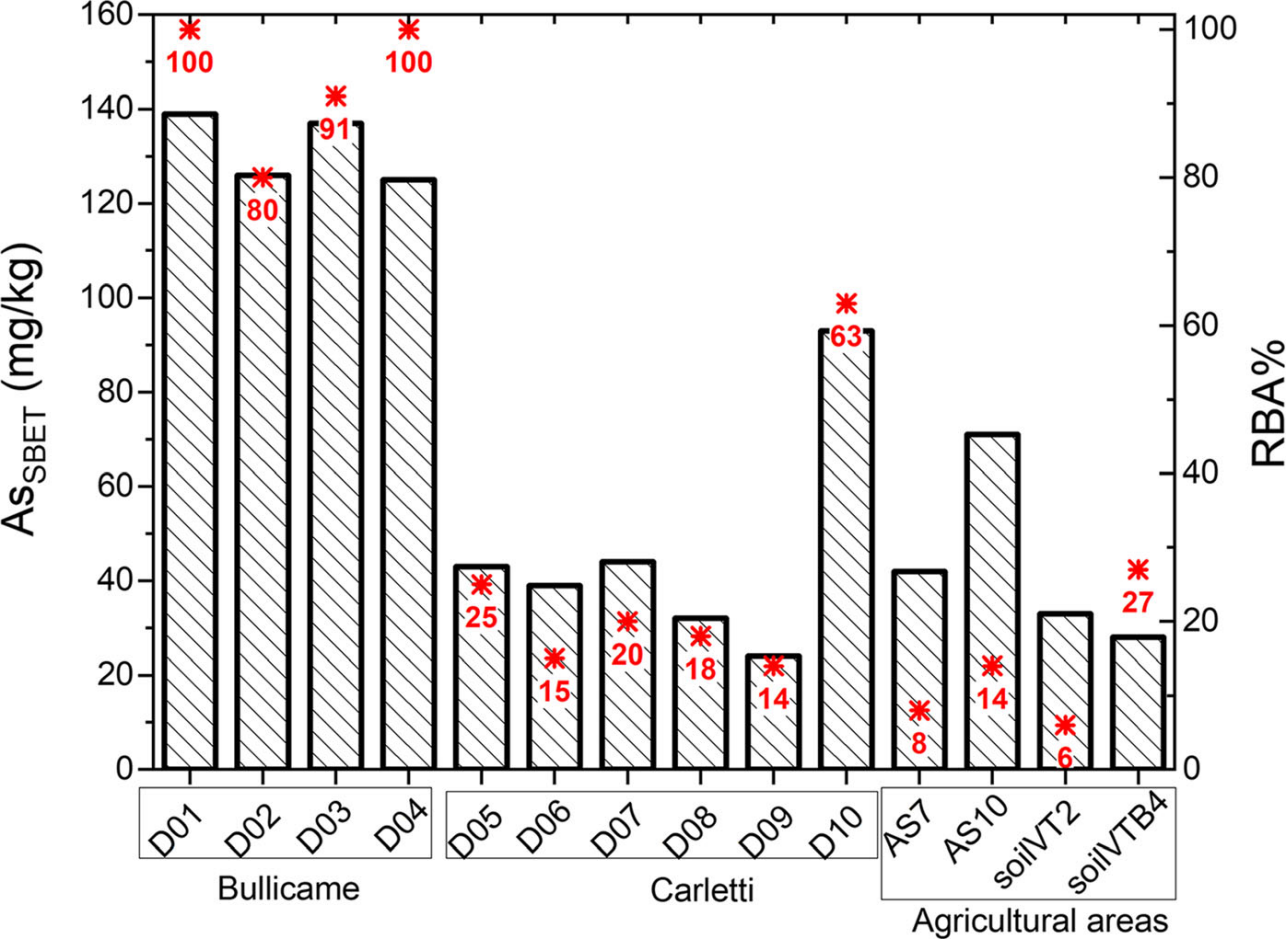
Bar chart of extracted As (mg/kg) during SBET (As_SBET_), and relative bioaccessible As (RBA,  %) values (numbers and asterisks in red)

Results of the bioaccessibility tests (As_SBET_ and RBA) are summarized in Fig. 2. Measured As_SBET_ concentrations range from 24 to 139 mg/kg As, and in some cases, it nearly corresponds to As_T-250_ concentrations, while RBA varied between 6 and 100%. Major differences in As_SBET_ concentrations and RBA are found among sampling sites. Soils from Bullicame show the highest As_SBET_ (125–139 mg/kg), corresponding to the highest relative bioaccessibility-RBA (80–100%); lower bioaccessible values (24–93 mg/kg As) are otherwise observed in soils from Piscine Carletti, with RBA of 15–63% (Fig. 2). It is important to note that the lowest percentages of RBA (7–27%) are found in agricultural soils, which display the highest As_T_ (130–528 mg/kg; Table 1).

## Discussion

### Arsenic source in soils of the Viterbo area

In the Viterbo area, a well-diffused geogenic As anomaly in groundwaters relates to the uprising of deep As-rich thermal waters (Angelone et al., 2009; Cinti et al., 2015, 2019; Vivona et al., 2007) that discharge at surface, precipitating travertine, dominantly composed by calcite (> 94 wt%) (Di Benedetto et al., 2011), and minor accessory phases (Fe_2_O_3_, TiO_2_, Al_2_O_3_ and MnO rarely exceeding 1 wt%). Arsenic repartition from solution to solid phase results in As enrichment in travertine, with up to ~ 280 mg/kg As found in fossil and new-forming deposits of the different plateau (Le Zitelle, Bullicame, and Piscine Carletti). Similar results were obtained by Dessau (1968), who documented up to 220 mg/kg of As in the travertine of Viterbo thermal springs (the exact location of the sampled spring is not given), and by Di Benedetto et al. (2011) in the travertine of Piscine Carletti spring (85–213 mg/kg As). Arsenic is often widespread in hot spring deposits (e.g., Webster and Nordstrom, 2003), but not many works investigated As in the associated travertine (Pentecost, 2005; Catelani et al., 2018). In addition to Costagliola et al. (2013), travertines with concentrations of As of hundreds to thousands mg/kg were documented in Iran (Hamidian et al., 2019; Khorasanipour & Esmaeilzadeh, 2015), Greece (Kampouroglou et al., 2017; Winkel et al., 2013), and Turkey (Dogan & Dogan, 2007), commonly associated with tectonically active areas, where the waning stages of Quaternary volcanic activity set up hydrothermal circulation at a basin scale, vehiculating emissions of CO_2_-rich fluids to the surface (Minissale et al., 2002). High concentrations of HCO_3_^−^, as those commonly observed at the thermal pools of Viterbo (Duchi et al., 1985; Di Benedetto et al., 2011), may indeed favor the leaching of As from the rock pile, represented in the study area by volcanic rocks (Casentini et al., 2010), during the fluid ascent (Anawar et al., 2004). Concentrations of 9–166 mg/kg As were indeed documented in rocks of the Vicano-Cimino system (Armiento et al., 2015; Casentini et al., 2010), together with As-bearing mineral phases (Della Ventura et al., 1991).

Soils main geochemical characteristics strictly correlate with the nature of parent rock (travertine vs volcanite) (Table S2). Arsenic contents (As_T-2000_; 24–56 mg/kg), except in sample BLS7, far exceed the national accepted Italian threshold limit (20 mg/kg) for recreational and residential use soils (D.L. 152/06). Our results expand the dataset of Zuzolo et al. (2020), who reported maximum As concentrations of 60 mg/kg in soils of the Viterbo area. Soils developed on travertines are particularly enriched in As when compared to those developed on the volcanic substratum (Mann–Whitney test, *p *≪ 0.05), suggesting that the As anomaly genetically relates to the formation of travertine plateau. For instance, the As is transferred to soils by dismantling the carbonate minerals.

**Fig. 3:**
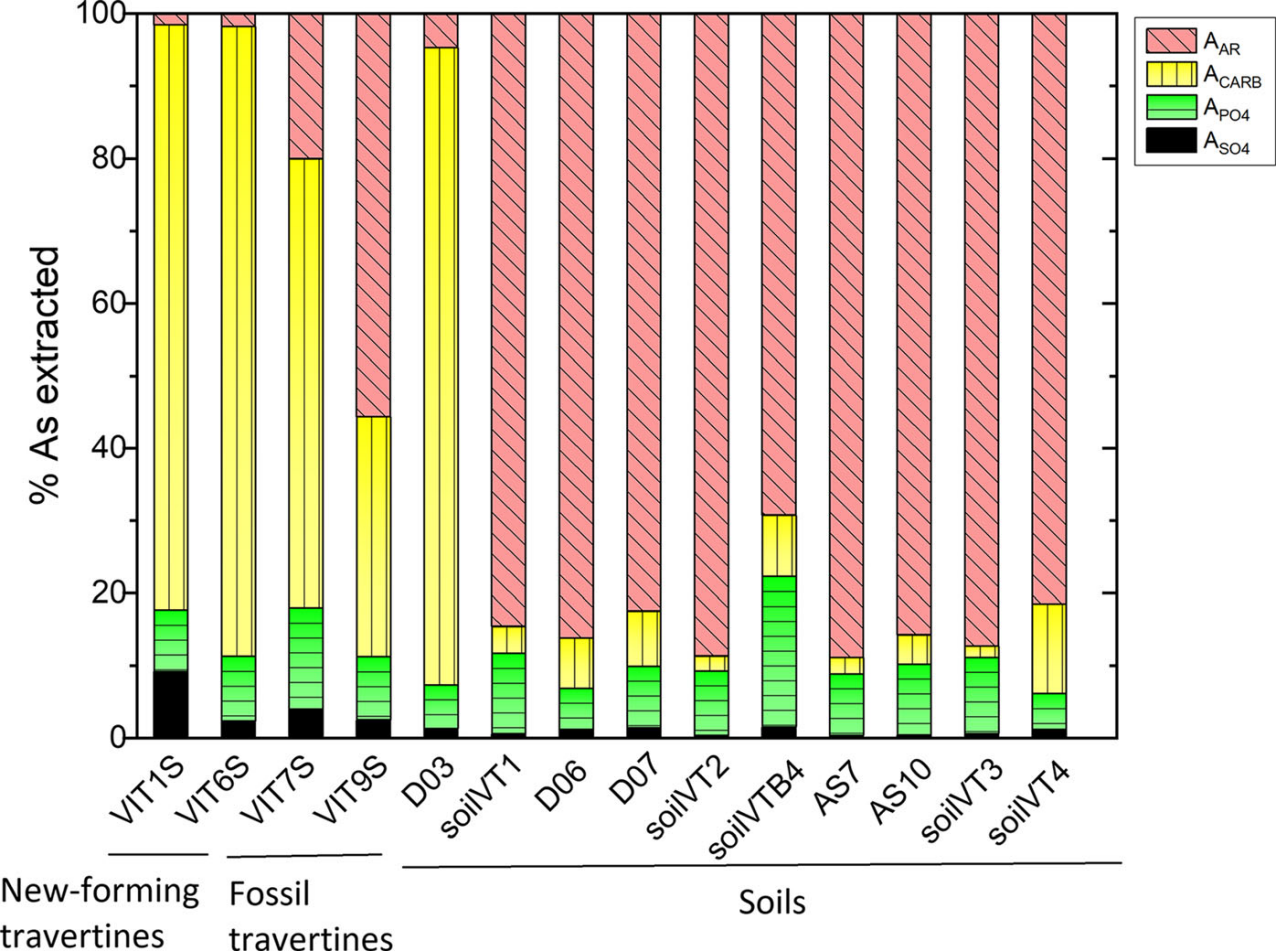
Percentages (normalized to 100% of extraction) of extracted As by SEC

Additional information on the processes controlling As distribution and partition in soils is provided by the sequential extraction procedures, specifically setup in this work to identify As speciation and leachability in samples characterized by high CaO contents. The carbonatic fraction hosts 81–88% of the total As (Fig. 3) in the new-forming travertines, and in soils at Bullicame (represented by sample D03), which are essentially a debris of the dismantled travertine rocks, and contain very abundant calcite (> 90 wt%). A specific and specifically sorbed As account for approximately 10% of the As budget, while the role of Fe(Mn)-oxyhydroxides in As sorption is negligible (~ 1–5% As is extracted in A_AR_). On the contrary, at Piscine Carletti and in the agricultural areas, where soils are poor in calcite (silicates are dominant in the mineralogical analysis) and well-developed (thickness ~ 50–60 cm, with abundant vegetation), only minor As (1–12%) is associated with the carbonate phase, and Fe(Mn)-oxyhydroxides have the prominent role for As trapping (82–89% of total As). It is worth to note that the A_PO4_ step accounts for up to 20% of the total As (Fig. 3), suggesting that the common employment of phosphate-based fertilizers in agricultural terrain may likely induce As mobilization from the soils due to the competitive PO_4_^3−^ for AsO_4_^3−^ exchange (Zeng et al., 2008). Fossil travertines collocate between the two end-members (i.e., high- and low-calcite soils), displaying intermediate percentage of As bound to calcite (33–63%; Fig. 3).

Iron oxides are well-known scavengers of As in the environment by establishing inner- and outer-sphere surface complexes (Giménez et al., 2007; Goldberg & Johnston, 2001), as well as As incorporation in the lattice structure. Besides Fe(hydr)oxides, laboratory experiments demonstrated that calcite efficiently retains As by surficial adsorption mechanisms (Sø et al., 2008) and/or structural incorporation (Alexandratos et al., 2007; Yokoyama et al., 2009, 2012). In natural travertines, As(III) can be incorporated in calcite through the substitution of AsO_3_^3−^ for CO_3_^2−^ (Bardelli et al., 2011; Di Benedetto et al., 2006). Recently, As(V) has been documented in the crystal lattice of calcite precipitating at Bullicame, while both As(V) and As(III) species were observed in biogenic calcites produced by the As-resistant bacterium *B. Licheniformis*, synthesized under laboratory condition on a solid medium (Catelani et al., 2018).

Data show that As-rich calcite dissolution from travertine likely controls As translocation in natural soils of the Viterbo area. Surface dissolution of calcite occurs during travertine diagenesis, when rocks are exposed to percolation of solutions undersaturated with respect to the carbonatic mineral, such as direct rainfall (Pentecost, 2005), and it is accompanied by Fe(Mn)- enrichment, likely suggesting Fe–Mn (hydr)oxides deposition. This is confirmed by major chemistry analysis of the fossil rocks, displaying lower CaO wt% and higher Fe_2_O_3_ and MnO wt% with respect to the travertine mud, and by SEC results showing progressively lower As recovery (both as absolute and percentage values) from the carbonatic step with increasing travertine age (Table 4; Fig. 3).

In conclusion, at Viterbo, calcite acts indeed only as a short-term trap for As, which is released by dissolution of the primary host, and finally transferred to Fe oxy(hydr)oxides during pedogenesis.

### Arsenic bioaccessibility and estimation of health risk

**Fig. 4:**
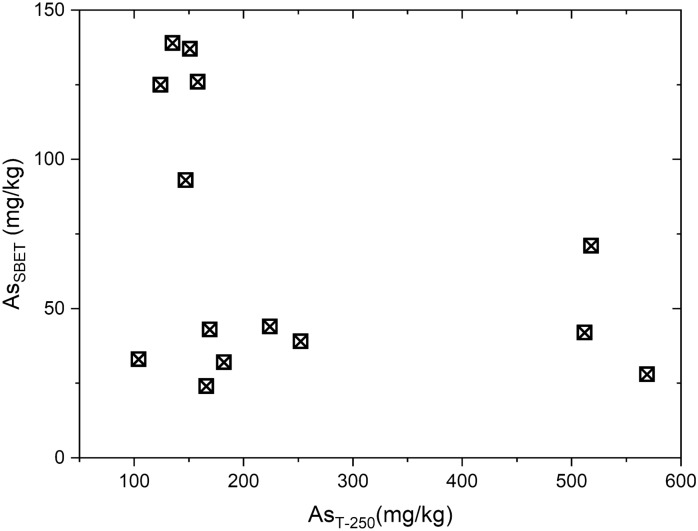
Bivariate plot showing (pseudo) total As in the 250 µm fraction (As_T-250_, mg/kg) and absolute bioaccessible As (As_SBET_, mg/kg)

In the Viterbo soils, As is distributed in different solid phases, which have different As bioaccessibility. Differently from other studies which found a linear correlation between bioaccessible concentrations of As and total concentrations in the soils (Hiller et al., 2018), total As contents in the studied soils are not explicative of As bioaccessibility (Fig. 4), which depends on the As mineral hosting phase. Relative bioaccessibility is variable among the investigated sites, reaching 80–100% in soils at Bullicame, and lowering to 6–63% at Piscine Carletti and in the agricultural areas (Fig. 2). It is our opinion that bioaccessibility is related to the presence of calcite, which easily dissolves in the gastric conditions simulated by the in vitro test. However, we suggest the opportunity of further investigations to increase the sampling number and to perform statistically significant tests.

The ranges of RBA of Bullicame soils are the highest documented in literature for soils where As bioaccessibility was determined by SBET. For example, up to 56.1% RBA was reported in playground soils of Bratislava, Slovakia (Hiller et al., 2018), or 46.3% in residential soils of Ambagarh Chowki block, India (Das et al., 2013). Significantly lower RBA was also documented in mine-impacted soils due to the presence of arsenopyrite and complex ferric arsenates, both hardly soluble in simulated gastric conditions (Drahota et al., 2017; Gamiño-Gutiérrez et al., 2013; Li et al., 2015). Similar to our study, high RBA (~ 80%) was observed in the playground soils of Madrid, where the presence of easily dissolved carbonate was hypothesized (Mingot et al., 2011), hence confirming the prominent role of carbonate minerals in controlling As bioavailability.

In this study, we specifically estimate the daily As intake, and the associated carcinogenic (CR) and non-carcinogenic (HQ) risks via ingestion of soils for different age groups of people (children and adults). Furthermore, other potentially relevant sources of As exposures connected to the thermal pools attendance are evaluated, like the As adsorption by dermal contact with soil and water, and thermal water ingestion for depurative purposes. The overall risk associated with the multiple exposure pathways is also evaluated. Data are summarized in Table 5.

**Table 5:** Results of the computed hazard quotient (HQ) and cancer risk (CR_skin_) for different age groups and exposure pathways with respect to thermal water and soil

Sample type	Exposure pathway	HQ				CR_skin_			
		Children		Adults		Children		Adults	
Thermal water	Ingestion	2.6	(88%)	2.3	(99%)	9.9E−05	(88%)	4.5E−04	(97%)
	Dermal contact	3.7E−03	(0%)	2.1E−03	(0%)	1.4E−07	(0%)	1.4E−07	(0%)
Soil	Ingestion (min–max)	5.8E−02	(12%)	3.1E−03	(1%)	2.3E−06	(12%)	2.7E−06	(3%)
		3.3E−01		1.8E−02		1.3E−05		1.6E−05	
	Dermal contact (min–max)	2.2E−03	(0%)	8.0E−05	(0%)	8.4E−08	(0%)	1.2E−08	(0%)
		1.2E−02		4.4E−04		4.6E−07		6.7E−08	
	HQ_TOT_	2.7–2.9		2.3		–		–	
	CR_skin,TOT_	–		–		1.0–1.1E−04		4.5–4.7E−04	

Double underlined numbers identify high carcinogenic and/or systemic risk (CR_skin_ > 1 × 10^−4^ or HQ > 1), while single underlined numbers indicate situation with moderate risk (CR_skin_ > 1 × 10^−5^). Percentages refer to the contribution of the exposure risk (HQ or CR) due to a single pathway scenario with respect to the total (HQ_TOT_, CR_skin, TOT_)

Based on the CR_skin,TOT_, the additional chance of developing a skin cancer during the lifetime due to the overall As exposure routes is in the range of 1.0–1.1 × 10^−4^ and 4.5–4.7 × 10^−4^ (Table 5) for children and adults, respectively. These values denote a potential high risk for population visiting the thermal pools, exceeding both the US EPA and Italian jurisdiction values of one additional case of cancer in one-million (1 × 10^−6^), which is used as a management goal for the risks posed by environmental contaminants. In detail, dermal exposure scenarios derived from water and soil contact are lower than the safer point (i.e., CR_skin,TOT_ = 1 × 10^−6^), being around 10^−7^–10^−8^ (Table 5), likely due to the low absorption of As through the skin. On the contrary, water ingestion is evidently the most relevant exposure route, accounting for 88–97% (Table 5) of the total CR_skin_ risk for children and adults, respectively. In the light of these data, consumption of thermal waters for depurative purposes, commonly practiced by local residents, should be strongly discouraged. On the other hand, As exposure due to soil ingestion is not entirely negligible, since it shows a moderate cancer risk for children (CR_skin_ = 1.3 × 10^−5^) and adults (1.5 × 10^−5^). This is especially relevant for children (12% of total cancer risk) due to the common mouth-to-hands activity during outdoor playing. Accordingly, the geographical distribution of As-related cancer risk due to soil ingestion highlights a moderate risk (1 × 10^−5^–1 × 10^−6^) in Italian central regions, like Latium (Zuzolo et al., 2020), as a consequence of geogenic As anomalies occurring in water and soils. In their work, Zuzolo et al. (2020) did not take into consideration bioaccessibility for risk calculation. However, we stress that it is a crucial step in exposure and risk analysis (US EPA, 2007a). The proposed method allows identifying the area at Bullicame as the one with the highest (1.1–1.5 × 10^−5^) cancer health risk, due to RBA approaching 100% of the total As budget in soils (Fig. 2).

The determined values of hazard quotient (HQ) indicate that As concentrations in soils via dermal or ingestion pathways or in water by dermal contact do not result in higher likelihood of non-carcinogenic health effects for people visiting the recreational thermal areas of Viterbo (HQ < 1; Table 5). However, a significant potential health risk (HQ = 2.3–2.6) for pathologies such as hyperpigmentation, keratosis, and possible vascular complications, associated with chronic exposure to As, occurs through thermal water ingestion.

Similarly, the lifetime consumption of As-contaminated tap water in the Viterbo region potentially exposes people to high skin cancer risk (1*10^−4^–10^−3^) and hazard risk (HQ = 4) (Zuzolo et al., 2020).

## Conclusions

Travertine and soils developed on volcanic and travertine substratum are investigated for As contents in and around the two most famous hot springs near Viterbo (Bullicame and Piscine Carletti), which host urban recreational areas highly frequented by local population and tourists. A wide geogenic As anomaly affecting both fossil and recently formed travertine is documented. In soils, As concentrations (42–528 mg/kg) largely exceeding Italian law limits spatially and genetically relate to the underlying travertine plateau. Sequential extractions and bioaccessibility test (SBET) are employed to elucidate the mineral phases controlling As bioaccessibility, and the processes that govern As distribution in the environment. SEC data indicate that in travertines and poorly developed soils, calcite is the primary mineral phase containing As (33–88% of the total As budget), while in well-developed soils As is mainly bound to Fe oxy(hydr)oxides (calcite only entrapped 1–12% of total As). Arsenic-rich calcite dissolution from new-forming travertine during diagenesis and pedogenesis is responsible for As partition in local soils and subsequent transfer to Fe oxy(hydr)oxides. Arsenic relative bioaccessibility is especially elevated in soils of the Bullicame area (80–100%), likely due to the presence of calcite, which is highly soluble in human gastric conditions, while it is low in agricultural soils (6–27%), where Fe oxy(hydr)oxides dominate. Arsenic exposure scenarios and associated risk analysis indicate that a moderate carcinogenic risk (CR_skin_ > 1 × 10^−5^) is present for adults and children by soil ingestion pathway. Nonetheless, the ingestion of contaminated water remains the principal exposure route for people attending the thermal pools. Therefore, we strongly suggest providing the thermal parks with public informative plaques discouraging people on the habit of thermal waters consumption.

## Supplementary Information

Below is the link to the electronic supplementary material.

Supplementary file1 (DOCX 28 kb)

## Data Availability

All data generated or analyzed during this study are included in this published article (and its supplementary information files). All data generated or analyzed during this study are included in this published article (and its supplementary information files).
