# Supplementary material for: Bioaccessible arsenic in soil of thermal areas of Viterbo, Central Italy: implications for human health risk
Source: Environ Geochem Health. 2021 Apr 21;44(2):465–85. doi: 10.1007/s10653-021-00914-1 (PMC8858286; doi:10.1007/s10653-021-00914-1)
Supplement: Supplementary file 1 — Supplementary file1 (DOCX 28 kb) [file 10653_2021_914_MOESM1_ESM.docx]

Supplementary information for the paper **Bioaccessible arsenic in soil of thermal areas of Viterbo, central Italy: implications for human health risk by Rimondi et al.**

Table S1. GPS coordinates of sample locations.

| **sample ID** | **GPS coordinates** | |
| --- | --- | --- |
| soilVT1 | 258507 | 4700807 |
| soilVT2 | 258409 | 4700723 |
| soilVT3 | 258251 | 4700684 |
| soilVT4 | 258073 | 4699821 |
| BLS 1 | 258731 | 4699523 |
| BLS 2 | 259239 | 4700515 |
| BLS 3 | 258954 | 4700918 |
| soilVTB1 | 257163 | 4700214 |
| soilVTB2 | 257462 | 4700658 |
| soilVTB3 | 258865 | 4705632 |
| soilVTB4 | 259304 | 4705254 |
| soilVTB5 | 259109 | 4705323 |
| AS3 | 257765 | 4697919 |
| AS7 | 257918 | 4695994 |
| AS10 | 258214 | 4701242 |
| D01 | 259188 | 4700557 |
| D02 | 259206 | 4700561 |
| D03 | 259218 | 4700602 |
| D04 | 259223 | 4700663 |
| D05 | 258544 | 4700782 |
| D06 | 258490 | 4700828 |
| D07 | 258486 | 4700841 |
| D08 | 258537 | 4700870 |
| D09 | 258546 | 4700935 |
| D10 | 258475 | 4700924 |
| D11 | 258475 | 4700924 |
| BLS4 | 256221 | 4700458 |
| BLS5 | 253904 | 4700921 |
| BLS6 | 253369 | 4700641 |
| BLS7 | 253458 | 4700280 |
| AS1 | 258045 | 4699222 |
| AS2 | 257956 | 4698819 |
| AS4 | 255647 | 4697310 |
| AS5 | 255758 | 4695276 |
| AS6 | 256814 | 4832546 |
| AS9 | 256696 | 4995838 |
| ZIT1 | 258209 | 4701290 |
| BL1 | 259191 | 4700608 |
| BL2 | 259197 | 4700610 |
| BL3 | 259224 | 4700261 |
| BL4 | 259239 | 4700571 |
| BL5 | 259243 | 4700568 |
| BL7 | 259250 | 4700564 |
| VT1s | 258507 | 4700807 |
| VT2s | 258507 | 4700807 |
| VT3s | 258507 | 4700807 |
| VT4s | 258507 | 4700807 |
| VT5s | 258507 | 4700807 |
| VT6s | 258507 | 4700807 |
| VT7s | 258507 | 4700807 |
| VT8s | 258409 | 4700723 |
| VT9s | 258251 | 4700684 |
| VT10s | 258507 | 4700807 |

Table S2

Main geochemistry of selected samples

| **sample ID** | **Sample type** | **LOI** | **Na_2_O** | **MgO** | **Al_2_O_3_** | **SiO_2_** | **P_2_O_5_** | **K_2_O** | **CaO** | **TiO_2_** | **MnO** | **Fe_2_O_3_** | **total** |
| --- | --- | --- | --- | --- | --- | --- | --- | --- | --- | --- | --- | --- | --- |
| soilVT1 | Trav soil | 10.56 | 0.55 | 1.72 | 17.72 | 54.82 | 0.22 | 2.97 | 2.87 | 0.91 | 0.61 | 7.05 | 100.0 |
| soilVT2 | Trav soil | 10.84 | 0.51 | 1.71 | 18.05 | 55.02 | 0.23 | 2.87 | 1.40 | 0.86 | 0.62 | 7.89 | 100.0 |
| soilVT3 | Trav soil | 11.64 | 0.39 | 2.02 | 17.86 | 54.12 | 0.25 | 2.83 | 2.02 | 0.73 | 0.38 | 7.76 | 100.0 |
| soilVT4 | Trav soil | 15.77 | 0.83 | 1.65 | 15.39 | 42.18 | 0.48 | 3.40 | 14.47 | 0.56 | 0.17 | 5.10 | 100.0 |
| AS3 | Trav soil | 18.86 | 0.23 | 0.72 | 17.15 | 31.75 | 0.13 | 2.66 | 18.23 | 1.10 | 0.13 | 9.06 | 100.0 |
| AS7 | Trav soil | 14.37 | 0.34 | 0.82 | 19.70 | 38.32 | 0.26 | 3.72 | 11.52 | 1.35 | 0.10 | 9.50 | 100.0 |
| AS10 | Trav soil | 23.99 | 0.27 | 0.84 | 10.22 | 24.89 | 0.47 | 2.36 | 27.31 | 0.75 | 0.32 | 8.49 | 99.91 |
| BLS5 | Vulc soil | 8.05 | 0.76 | 1.19 | 19.92 | 48.42 | 0.18 | 5.78 | 3.84 | 1.17 | 0.29 | 10.4 | 100.0 |
| BLS6 | Vulc soil | 5.99 | 1.08 | 1.09 | 21.38 | 48.93 | 0.28 | 5.86 | 3.98 | 1.08 | 0.35 | 9.98 | 100.0 |
| BLS7 | Vulc soil | 8.56 | 1.1 | 1.27 | 18.12 | 48.24 | 0.32 | 7.12 | 5.8 | 1.01 | 0.24 | 8.21 | 100.0 |
| AS2 | Vulc soil | 7.51 | 1.17 | 0.98 | 20.45 | 48.87 | 0.22 | 6.03 | 3.57 | 1.18 | 0.25 | 9.77 | 100.0 |
| AS9 | Vulc soil | 7.6 | 0.9 | 1.67 | 20.57 | 46.99 | 0.26 | 4.2 | 5.18 | 1.11 | 0.22 | 11.3 | 100.0 |
| VT1s | New-form trav | 42.75 | 0.06 | 0.98 | 0.60 | 1.12 | <0.01 | 0.05 | 54.34 | 0.01 | 0.02 | 0.07 | 100.0 |
| VT2s | New-form trav | 42.56 | 0.05 | 1.03 | 0.50 | 1.06 | <0.01 | 0.05 | 54.68 | 0.01 | 0.02 | 0.05 | 100.0 |
| VT3s | Fossil trav | 42.21 | 0.06 | 0.99 | 0.58 | 1.44 | 0.01 | 0.10 | 54.42 | 0.01 | 0.01 | 0.15 | 100.0 |
| VT4s | New-form trav | 42.66 | 0.05 | 1.10 | 0.10 | 0.29 | <0.01 | 0.04 | 55.70 | <0.01 | 0.01 | 0.04 | 100.0 |
| VT5s | New-form trav | 42.87 | 0.03 | 1.06 | 0.07 | 0.37 | 0.01 | 0.03 | 55.53 | <0.01 | 0.02 | 0.01 | 100.0 |
| VT6s | New-form trav | 42.23 | 0.05 | 1.04 | 0.22 | 0.6 | 0.03 | 0.06 | 55.66 | 0.01 | 0.02 | 0.09 | 100.0 |
| VT7s | Fossil trav | 43.60 | 0.03 | 0.61 | 0.35 | 0.84 | 0.05 | 0.05 | 54.02 | 0.01 | 0.29 | 0.19 | 100.0 |
| VT8s | Fossil trav | 41.83 | 0.04 | 0.45 | 0.33 | 6.22 | 0.03 | 0.06 | 50.68 | 0.01 | 0.04 | 0.31 | 100.0 |
| VT9s | Fossil trav | 42.45 | 0.02 | 0.78 | 1.06 | 3.05 | 0.05 | 0.14 | 51.17 | 0.04 | 0.15 | 1.06 | 100.0 |
| VT10s | New-form trav | 43.05 | 0.04 | 0.61 | 0.49 | 1.23 | 0.05 | 0.07 | 54.35 | 0.01 | 0.01 | 0.11 | 100.0 |
